# Ensuring Timely Pulmonary Follow-up after an Inpatient Asthma Hospitalization: A Quality Improvement Initiative

**DOI:** 10.1097/pq9.0000000000000815

**Published:** 2025-05-14

**Authors:** Leela Chandrasekar, Hollie Schaffer, Sanjiv Godse, Matthew Grossman, Laura Chen, Eliaz Brumer

**Affiliations:** From the * Department of Pediatrics, Section of Respiratory, Allergy-Immunology, and Sleep Medicine, Yale University, New Haven, Conn.; † Department of Pediatrics, Section of General Pediatrics, Yale University, New Haven, Conn.

## Abstract

**Introduction:**

Timely outpatient follow-up after hospitalization for asthma exacerbation is essential for ongoing management and preventing future episodes. We identified significant variability in scheduling postdischarge pulmonology follow-up, leading to inconsistent care. This quality improvement initiative aimed to ensure at least 90% of patients admitted for an acute asthma exacerbation who had been seen by the pulmonology team scheduled for an outpatient pulmonary follow-up with an 80% attendance rate within 45 days of hospital discharge.

**Methods:**

A multidisciplinary team developed 3 key drivers. Key interventions included developing standardized asthma care guidelines and ensuring timely pulmonary consultation for all patients admitted to the pediatric intensive care unit with asthma exacerbation. The pulmonary team was also notified of patients previously seen by the department who were admitted to the floor for asthma exacerbation. The outcome measures included the percentage of patients admitted with asthma exacerbation scheduled for pediatric pulmonology follow-up appointments within 45 days and the percentage attending those appointments.

**Results:**

The percentage of scheduled appointments increased from 58.7% to 97.3%, and the appointment attendance rate improved from 45.3% to 85.2%. A retrospective review 3 years after the project’s implementation showed sustained improvement, with 93% of appointments scheduled and 82.7% attended.

**Conclusions:**

Scheduling pulmonary follow-up appointments before discharge and using active reminders with immediate rescheduling of cancelations improved outpatient visit attendance. Further research is needed to confirm whether timely follow-up enhances asthma control and reduces readmissions.

## INTRODUCTION

Asthma is a chronic respiratory illness characterized by wheezing, cough, and chest tightness, affecting about 14% of children worldwide.^1^ Acute asthma exacerbations are a significant cause of pediatric morbidity and mortality. Children who experience severe exacerbations requiring hospitalization are at the highest risk for future severe episodes, progressive lung function decline, and asthma-related mortality.^2,3^ Following an exacerbation, evaluating for triggers and assessing what interventions can improve asthma control and reduce future exacerbations is essential.^4–6^

At the time of project initiation, the process for scheduling posthospitalization pulmonology follow-up was nonstandardized and inconsistent. Fewer than 60% of patients with asthma exacerbations were scheduled for follow-up, raising concerns about continuity of care and asthma control after discharge. This initiative aimed to optimize pediatric pulmonology outpatient follow-up after acute exacerbations for children known to our division who required admission and all children admitted to the pediatric intensive care unit (PICU) for asthma exacerbation.

Similar interventions have improved outcomes. Ruffner et al^6^ showed that an allergy clinic–based intervention with proactive scheduling increased follow-up attendance and reduced 30-day readmissions. Likewise, Zorc et al^7^ found that scheduling follow-ups during pediatric emergency department (ED) visits improved follow-up rates with pediatricians. Additionally, Backer et al^8^ demonstrated that structured asthma care incorporating scheduled follow-ups and systematic management—guided by Global Initiative for Asthma principles^9^—was associated with improved adult outcomes, highlighting the potential value of a standardized follow-up process.

Our goal was to ensure that at least 90% of children admitted for asthma exacerbation who were either previously followed by our division or required a PICU admission were scheduled for pediatric pulmonology follow-up within 45 days of hospital discharge and at least 80% of all children identified, regardless of whether a follow-up appointment was scheduled, attended these appointments. This project aimed to address the identified gap in timely postdischarge follow-up care by optimizing timely specialty follow-up, which is essential for reassessing asthma triggers, refining management plans, identifying modifiable risk factors, and ultimately preventing future exacerbations.

## METHODS

### Context: Baseline (January 2020–July 2020)

This quality improvement (QI) project was conducted from January 2020 to July 2022 at Yale New Haven Children’s Hospital, an urban medical center with approximately 3,000 asthma ED visits and an average of 550 admissions annually during the project timeline.

The pediatric pulmonary clinic primarily manages moderate to severe or difficult-to-treat asthma cases and annually follows around 2,000 asthma patients. The clinic offers extended resources, including dedicated asthma education, pharmacist support for medication access and management, expertise in biologic therapies, and advanced pulmonary function testing when indicated. During the study period, the pulmonary division comprised 7 faculty members, 2–4 clinical fellows annually, and 1 nurse practitioner. Approximately 14 half-day weekly clinics are devoted to general pulmonary care, with asthma patients representing the majority.

All patients admitted for asthma exacerbation who had been seen at least once by our pediatric pulmonology department 3 years before their current asthma exacerbation admission or were newly consulted during the current hospitalization were included. Patients with comorbidities, such as chronic lung disease, severe scoliosis, and congenital heart disease, were excluded, as their chronic condition may have been the primary cause of their respiratory admission rather than a pure asthma exacerbation. The patient population remained consistent across the baseline, intervention, and postintervention phases, except for the decrease in census during the COVID-19 pandemic. We analyzed data at the aggregate level, and individual patient-level demographic or clinical data (such as race/ethnicity or asthma severity) were not collected for this project.

As there was no standardized approach to tracking asthma admissions, our hospital’s data analytics department developed a dataset/list at the start of the project to identify patients likely admitted due to asthma exacerbation. This dataset, generated from Yale New Haven Health System’s electronic medical records (Epic Systems, Verona, Wis.), was updated automatically daily.

This list included patients labeled with any International Classification of Diseases, 10th revision codes suggestive of respiratory disorders [eg, reactive airway disease (J45. 909), status asthmaticus (J45. 902), asthma exacerbation (J45. 901), etc.]. The list was then manually screened by reviewers (E.B. and S.G.) to ascertain whether our department followed the identified patients due to asthma or had been consulted during the index admission and not due to other respiratory comorbidities. Patients identified with comorbidities that met exclusion criteria or with an admission diagnosis unrelated to asthma exacerbation were removed from the list. The QI team monitored the dataset biweekly throughout the project. In total, medical records for 201 patients were reviewed.

Patients were placed on the timeline based on their discharge date. For each month, 2 metrics were calculated: (1) the percentage of patients discharged after an asthma exacerbation who had a follow-up appointment scheduled within 45 days of discharge and (2) the percentage who attended a follow-up appointment within 45 days of discharge, regardless of whether it had been scheduled.

### Intervention (August 2020–January 2021)

Despite limited baseline data, the intervention began in August 2020, prompted by internal findings and US News & World Report benchmarks^10^ showing below-average follow-up rates.

**Fig. 1.**
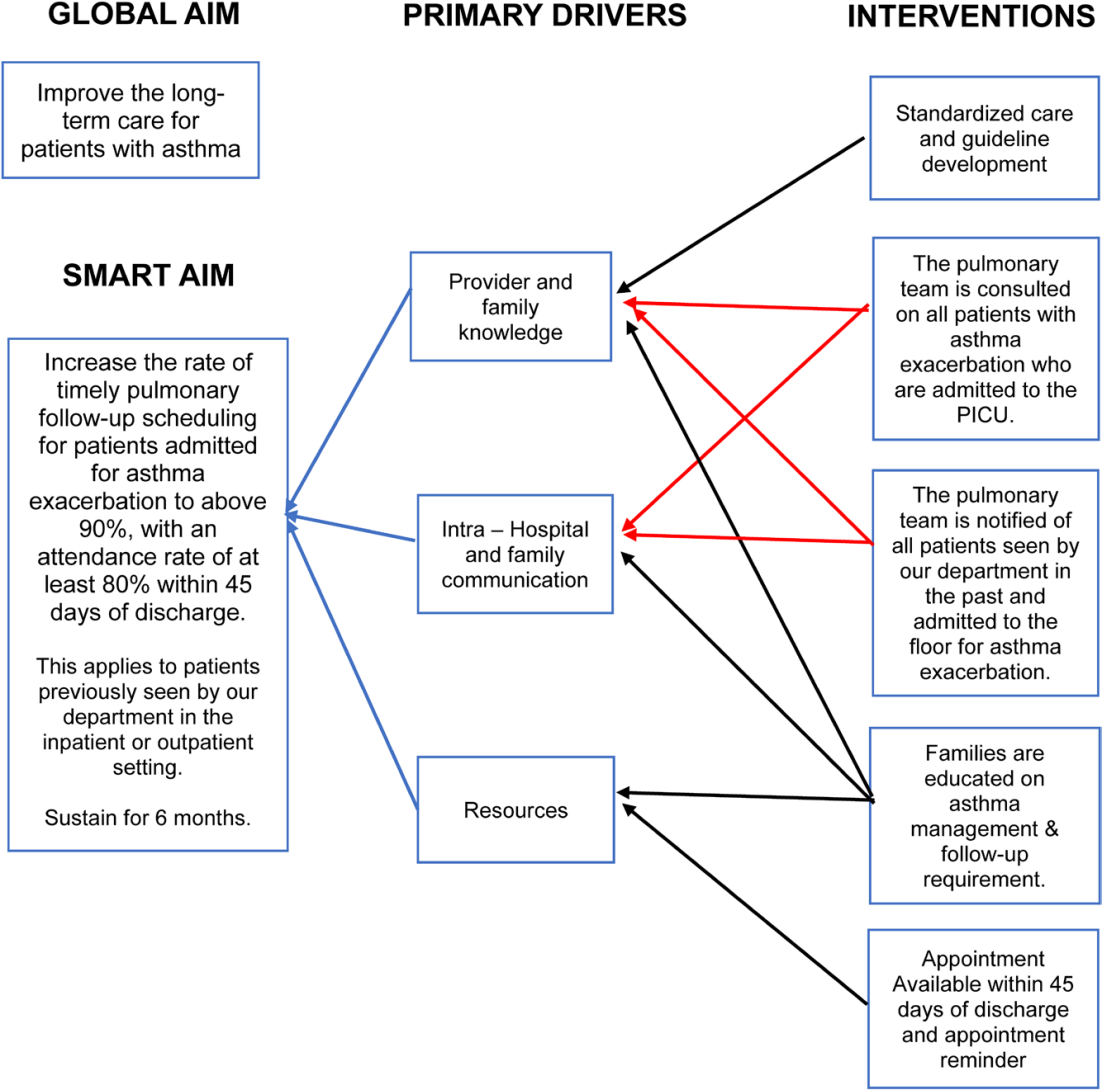
Key driver diagram.

During the intervention phase, a multidisciplinary team consisting of pediatric pulmonologists, nurses, and the pediatric pulmonary administrative team was established. The QI team identified 3 key drivers: (1) provider and family knowledge, (2) communication between the pediatric pulmonology team, pediatric team, PICU team, and families, and (3) available resources that could impact timely pulmonary follow-up. During the project, 5 interventions were implemented (Fig. 1) to increase the rate of scheduled and completed appointments for patients with asthma.

#### Provider and Family Knowledge

##### Standardized Care and Guideline Development

**Fig. 2.**
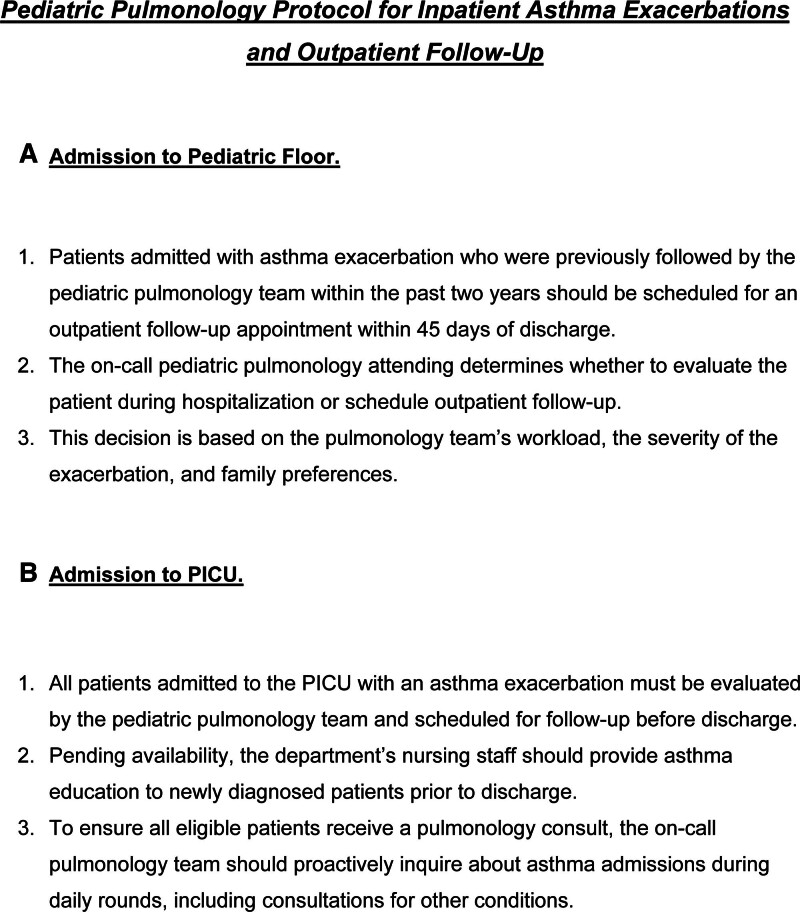
Pediatric pulmonology protocol for inpatient asthma exacerbations and outpatient follow-up.

The Department of Pediatric Pulmonology thoroughly reviewed the existing literature^9,11–13^ on managing asthma and recommendations for outpatient follow-up after admission for asthma exacerbations. The team developed a standardized guideline (Fig. 2), requiring all pediatric patients previously seen by the department during the 3 years before their current admission and admitted for asthma exacerbation to have a follow-up within 45 days of discharge. Mandatory inpatient pediatric pulmonology consultations were implemented for all pediatric patients admitted to the PICU for asthma exacerbations before discharge.

Throughout the project (quarterly during the baseline and intervention periods and approximately every 6 mo during the later phases), QI meetings were conducted within the pediatric pulmonology to review outcomes and reinforce adherence to the intervention protocols. These meetings focused on maintaining effective communication with the pediatric department and ensuring that the team was notified when patients previously followed by the department were hospitalized with asthma exacerbations. Daily collaboration with the PICU team was also prioritized to identify and confirm asthma-related admissions.

#### Intra–Hospital and Family Communication

##### The Pulmonary Team Is Consulted on All Patients with Asthma Exacerbation Who Are Admitted to the PICU

Mandatory formal pediatric pulmonology consultations were initiated through EPIC for all patients in the PICU before transfer to the general inpatient floor to improve care for pediatric patients with asthma exacerbations. This initiative was established through a collaborative effort between the Department of Pediatric Pulmonology and the PICU. The PICU team included the PICU attending physicians, the charge advanced practice registered nurses, and the PICU fellows. Additionally, the pediatric pulmonary team on-call was expected to proactively inquire about asthma admissions during daily interactions, even when consulting on other cases, to ensure that all eligible patients received a pulmonology consult.

All identified patients, whether new or previously known to the department, received a comprehensive evaluation. Their treatment plans were optimized with individualized asthma education by the division’s nurses, and discharge instructions were provided to their families. Before discharge, follow-up appointments with the pulmonology team were scheduled by the administrative team, and the importance of attending these appointments was emphasized to the parents or guardians.

##### The Inpatient Pediatric Team Notifies the Pulmonary Team Of All Patients Seen by Our Department in the Past 3 Years and Admitted to the Floor for Asthma Exacerbation

The pulmonary team worked closely with the inpatient pediatric team, primarily hospitalists, chief residents, and senior residents, to optimize asthma inpatient care and emphasize the importance of timely outpatient follow-up. When a patient previously evaluated by the department was admitted to the general pediatric floor for asthma exacerbation, a member of the inpatient pediatric team, typically an intern, notified the on-call fellow, and the attending pulmonologist determined whether a consultation was necessary. Upon notification, the patient’s prior history and current management were reviewed to ensure consistency with their individualized asthma care plan.

The decision to see a patient during hospitalization depended on the worklod, the severity of the patient’s asthma exacerbation, and family requests. The pulmonary administrative team handled the scheduling of follow-up appointments as requested by the pulmonary fellow. If a notification was not made, and the patient was not scheduled for a follow-up appointment, they were still included in the denominator of the project metrics.

##### Families Are Educated on Asthma Management and Follow-up

During the formal pediatric pulmonology consultation, the medical team engaged with families, providing education about asthma management. This included detailed discussions on the necessity of outpatient follow-up and informing them about the heightened risk of hospital readmission. The initial follow-up visit was scheduled within 45 days of discharge, with subsequent appointments determined by patient characteristics and the pediatric pulmonologist’s decision.

#### Resources

##### An Available Appointment within 45 Days of Discharge and Appointment Reminder

A 45-day follow-up window was selected to provide sufficient time for scheduling, considering clinic capacity and family availability while maintaining timely postdischarge evaluation. Before hospital discharge, the pulmonary team, with the support of administrative staff, scheduled follow-up appointments for patients. The administrative team contacted families to schedule follow-up appointments, prioritizing the most convenient time and the closest clinic location for each family. Efforts were made to schedule each patient with their primary pediatric pulmonology provider whenever an appointment was available within the 45-day follow-up window. If no availability existed, the patient was scheduled with another provider, most often in the pulmonary fellow’s clinic. Telehealth appointments were also utilized to facilitate follow-up, particularly during the pandemic.

Additionally, the administrative team actively reminded families about their appointments. These reminders were made via telephone calls, during which staff also informed families of the need to arrive early for pulmonary function testing when applicable. The hospital’s centralized call center sent SMS reminders in English or Spanish (based on the preferred language recorded in the electronic medical records) a few days before the appointment, also requesting confirmation. If a patient needed to cancel their appointment, efforts were made to reschedule at the next available time convenient for the family, with a goal of maintaining the follow-up within 45 days to meet the care objectives.

##### Clinic Hours Expanded

Initially, no additional resources were allocated, as asthma exacerbations remained low during the pandemic. However, additional accommodations were implemented as patient volume increased for all pulmonary clinics. These included the introduction of a monthly Saturday morning clinic, an evening clinic held once or twice per month, and later urgent add-on clinics scheduled approximately once a week as needed to manage the rising demand for follow-up appointments.

### Measures and Analysis

The primary outcomes were (1) the percentage of asthma patients followed by our department who were scheduled for a follow-up appointment within 45 days of hospital discharge and (2) the percentage of all patients with asthma exacerbation, regardless of whether they had a scheduled appointment, who successfully attended a follow-up visit within 45 days of hospital discharge. Medical records from the patient list were reviewed twice a month to ensure that appointments were made and no patients with asthma were missed. Statistical Process Control charts were used to track the progress. Special cause variation was defined as the occurrence of at least 8 consecutive points above or below the centerline. The charts were developed using Microsoft Excel QIMacros (KnowWare Int, Denver, Colo.).

### Long-term Outcomes (June 2023–April 2024)

Although active project monitoring concluded in December 2022, a retrospective review of patient data from June 2023 to April 2024 was conducted using the same database and inclusion criteria established during the original project. This review aimed to assess whether improvements in scheduling and attendance were sustained despite no active interventions during this period. The list, consisting of 110 patients, was manually screened by reviewers (L.C. and H.S.), averaging 10 minutes per patient. The review focused on determining whether a follow-up appointment was scheduled and if the patient attended the appointment within 45 days of discharge.

### Ethical Considerations

This project was designed to improve clinical practice and processes at our institute. Based on our hospital’s QI criteria, it was exempt from institutional review board review.

## RESULTS

During the baseline period (January 2020–July 2020), 70 patients with International Classification of Diseases, 10th revision codes suggestive of asthma exacerbation were identified, which our pediatric pulmonology department followed. Nineteen patients were removed as they had other comorbidities that could explain their respiratory exacerbation and admission despite being categorized as asthma exacerbation. As a result, 51 patients were included in the baseline, 28 during the intervention period, and 103 during the follow-up period.

**Fig. 3.**
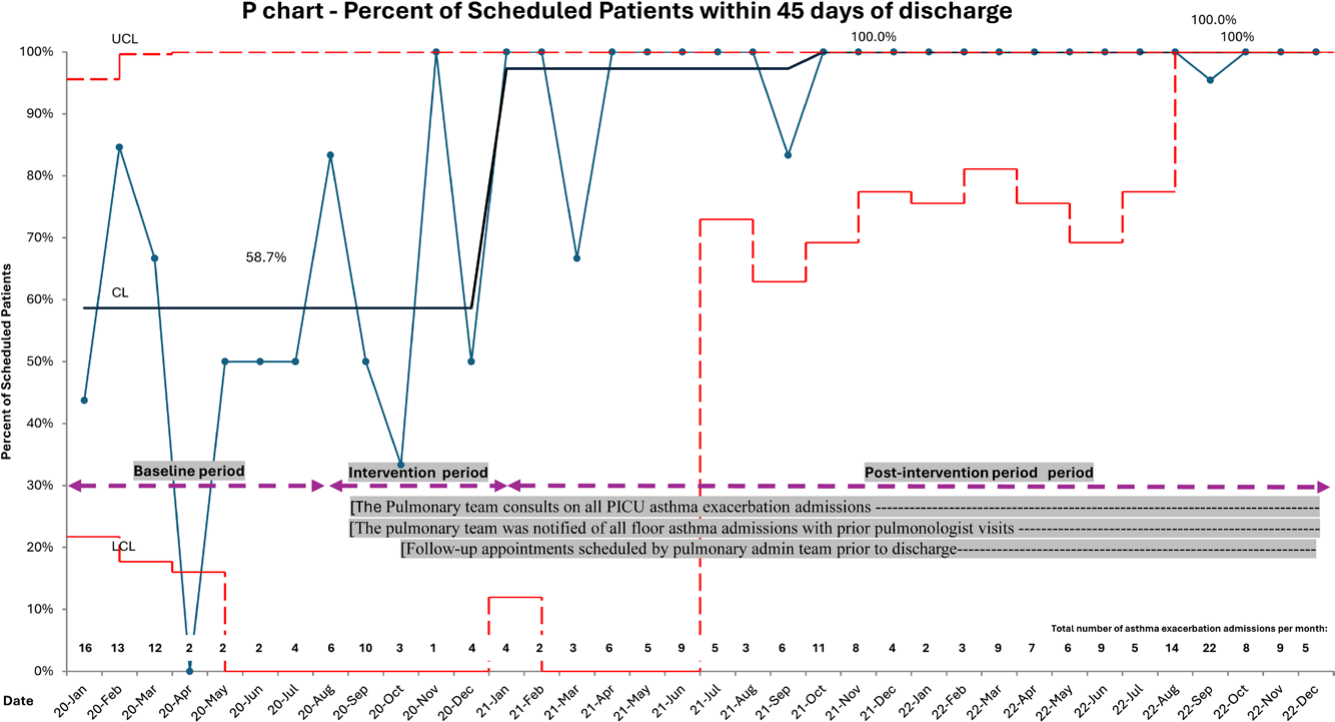
Percent appointments scheduled within 45 days of discharge for patients with asthma exacerbation. Statistical Process Control chart, January 2020 to December 2022. CL, center line; LCL, lower control limit; UCL, upper control limit.

**Fig. 4.**
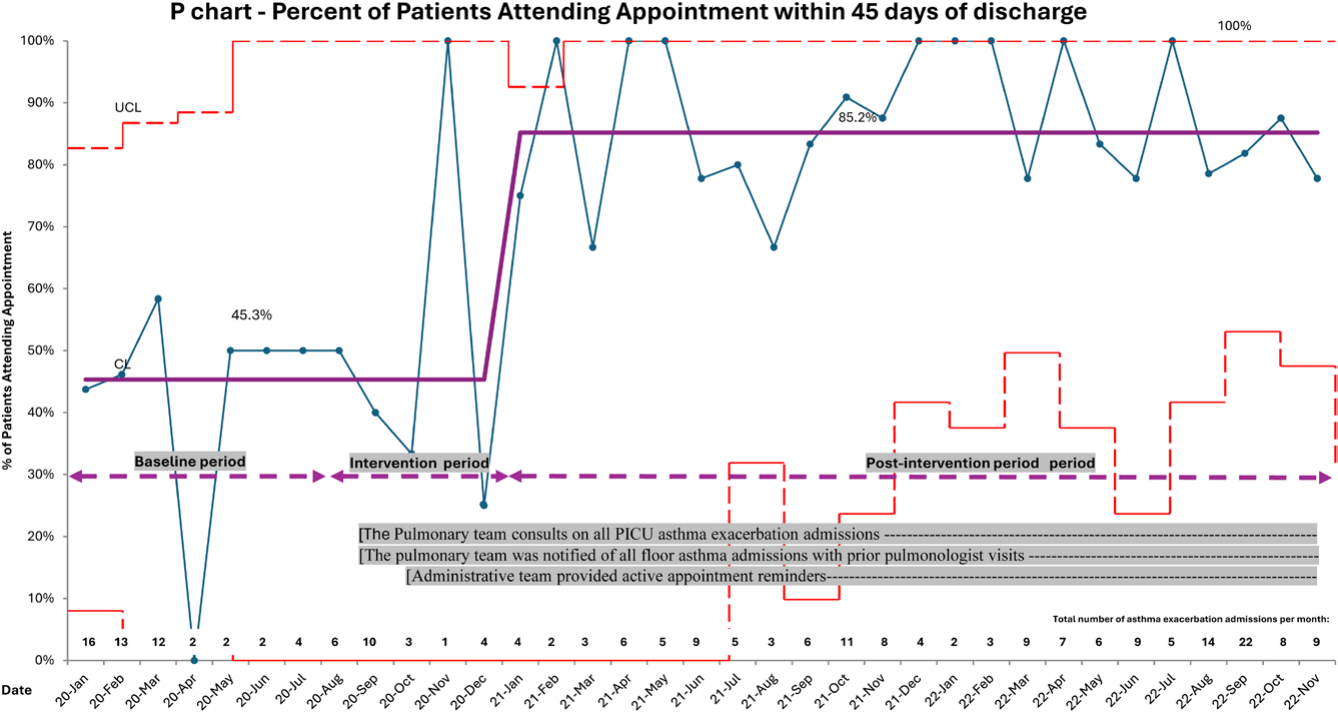
Percent attendance of scheduled appointments within 45 days for asthma exacerbation. Statistical Process Control chart, January 2020 to December 2022. CL, center line; LCL, lower control limit; UCL, upper control limit.

During the baseline period, 58.7% were scheduled for follow-up 45 days after discharge. Special cause variation was achieved in January 2021. After the interventions were completed, 97.3% were scheduled for follow-up 45 days after discharge (Fig. 3). A secondary special cause variation was achieved in October 2021 with a 100% scheduling rate. During the baseline period, the overall attendance rate remained at 45.3%. With the implementation of the interventions, special cause variation was achieved in January 2021, with an attendance rate of 85.2% for patients with asthma (Fig. 4).

The long-term outcomes from June 2023 to April 2024 revealed a scheduling rate of 93.6% and an attendance rate of 82.7% among the 110 patients evaluated (data not shown).

## DISCUSSION

The study identified significant variability in scheduling follow-ups for pediatric patients admitted with asthma exacerbations, which risked inconsistent postdischarge care and suboptimal asthma management. Baseline data reflected this challenge, revealing that only 58.7% of patients were scheduled for follow-up, and only 45.3% attended within 45 days of discharge. This finding aligns with previous studies indicating that a substantial proportion of patients do not receive timely follow-up after hospitalization for asthma.^14–17^

Implementing 5 targeted interventions addressing 3 key drivers resulted in an increase in the percentage of patients scheduled for follow-up appointments to 97.3%, with an attendance rate of 85.2%. The scheduling rate reached 100% by October 2021 and was notably sustained for nearly 3 years following the intervention.

Our approach focused on embedding systemic changes within the inpatient care process, including mandatory pulmonary consults for PICU patients and scheduling follow-ups before discharge. This proactive, multidisciplinary model may explain the higher follow-up attendance rates observed, as it minimizes reliance on families to initiate scheduling postdischarge and ensures comprehensive asthma education and care planning occurs during hospitalization.

Importantly, the literature suggests that specialist follow-up may yield better outcomes than primary care alone.^18^ Children enrolled in a structured asthma care model, including in-person, multidisciplinary, and telehealth approaches, had improved lung function, fewer school absences, and reduced emergency visits compared with those in usual care^17,19,20^ Similarly, follow-up by asthma specialists has been associated with reduced readmissions and healthcare utilization.^18^ These findings support a model that prioritizes timely follow-up by pediatric pulmonologists, leveraging the specialized expertise and resources available within the pulmonology clinic.

Over the 3 years following the project’s initiation, the department underwent significant transitions in staffing and organizational structure. Pediatric residents who had participated in the early phases of the project completed their training, fellows from both our division and the PICU graduated, and the administrative team experienced turnover. Despite these changes, the intervention’s effectiveness endured. A long-term review of the project, conducted from June 2023 to April 2024, demonstrated continued success, with 93.6% scheduling and 82.7% attendance rates. These outcomes persisted despite increased patient volumes, validating the resilience of the intervention and highlighting the value of building adaptable processes that can accommodate fluctuations in demand.

We believe the resilience of these outcomes can be attributed to several factors. First, embedding the intervention into routine practices for attendings on the floor and in the PICU ensured continuity despite staff turnover. Second, regular presentations and discussions of the project within the division reinforced its importance, fostering a culture of accountability and commitment to the established processes. These mechanisms ensured that the original goals for scheduling and attendance rates were upheld, even as the system faced inevitable challenges associated with changes in personnel and increasing patient volumes.

## LIMITATIONS

The study had 2 notable limitations. First, it was limited to a single center, which may impact the generalizability of the findings to other healthcare settings. Second, the study did not assess ED/admission return rate, which is essential to understanding the full impact of the interventions. Future studies should examine whether improved follow-up rates lead to reductions in exacerbations and healthcare utilization over time.

Additionally, although we did not use a standardized method to identify all PICU asthma admissions, which may have led to missed patients and an underestimation of follow-up metrics, we mitigated this limitation through consistent communication with the PICU team. Nonetheless, implementing structured alerts may help reduce the risk of missed patients in future iterations.

Moreover, although multiple formal PDSA cycles were not conducted, ongoing multidisciplinary meetings played a key role in sustaining the intervention. These meetings served as a platform for reviewing progress, reinforcing key practices, and promoting continued collaboration among teams, which likely contributed to the durability of the outcomes.

Finally, although balancing measures were not directly analyzed, their relevance in interpreting the outcomes warrants attention. Variables such as the availability of pulmonology appointments and the capacity of the pulmonology team to handle consult demands may have influenced the results. These considerations could pose challenges to replicating and scaling the intervention. Although not quantified in the study, they represent critical factors that should be addressed in future investigations to enhance the comprehensiveness and applicability of QI initiatives.

## CONCLUSIONS

Our findings highlight the effectiveness of scheduling follow-up appointments before discharge and implementing active reminder systems to improve outpatient visit attendance for children with asthma. These interventions demonstrated sustainability over a 3-year period and provided a framework for consistent postdischarge care despite fluctuations in patient volumes and staffing changes. Although additional research is needed to determine the impact on asthma control and readmissions, this project underscores the value of structured, adaptable QI initiatives in enhancing continuity of care and patient outcomes. This model can inform future efforts to improve chronic disease management in pediatric populations by addressing operational challenges and fostering a culture of accountability.
